# Introducing the “Microbiomes and Social Equity” Special Collection

**DOI:** 10.1128/msystems.00806-22

**Published:** 2022-08-29

**Authors:** Sue Ishaq, Jack Gilbert

**Affiliations:** a School of Food and Agriculture, University of Maine, Orono, Maine, USA; b Department of Pediatrics and Scripps Institution of Oceanography, University of California San Diego, La Jolla, California, USA

## Abstract

The Microbes and Social Equity (MSE) working group formed in early 2020 out of a need to place microbiome research in a social and political context so as to be able to address health inequities that are rooted in microbiology. A special collection of articles was commissioned by the MSE group to introduce these concepts to a wider audience.

## EDITORIAL

There is no aspect of your life which does not involve microbes, from the ones that assist in digestion to the ones that helped you create that sourdough bread that you shared on social media. Microbes are the basis for biodiversity on this planet, and they quietly enrich our lives. Most of the time, we don’t even remember that microbes are always with us, but even encounters as incidental as trading a few microbes with someone we walk by on a narrow sidewalk can affect us. Microbiome research has immersed itself in studying the ways that humans encounter, recruit, or battle microbes, and through this exploration we have come to appreciate how much context matters to the form and function of that microbial community.

The environments in which people spend their time, the decisions they make, and the decisions that are made for them or against them may all influence the microbes they encounter. Similarly, there is no aspect of your life which does not involve social policy, including the foods you have, or are denied, access to and the safety standards applied to your food or drinking water; air quality and exposure to pollutants; which animals you own, how many, and for what purpose; bodily autonomy in medical decisions; and even the amount of natural habitat around you and which biodiversity it contains. All of these can directly impact your encounters with microbes.

It stands to reason that to understand the connections between humans and microbiomes, we must view this context through the lens of social systems and policies which enable or restrict our choices and thereby our agency in recruiting beneficial microbes or establishing balanced host-microbe relationships. To understand the differences in microbiomes across peoples and places, and to make meaningful recommendations to improve health, we need to acknowledge the social inequity which restricts access to public resources that we all have a right to in principle, if not always in practice. And as human bodies and health exist in a gendered, classed, racialized, or colonized manner in the world, human, microbial, and environment interactions are shaped by those forces. Microbiomes are not immune to politics.

MSE has connected global microbiological, social, political, building, and computational scientists, along with anthropologists, architects, ecologists, wildlife biologists, bioethicists, psychologists, and philosophers, to foster these conversations and to bring fresh perspective to the transformative early research on these concepts which implicated socioeconomic disparities as a factor in microbiome disparity.

For this collection, we invited contributions from researchers working across disciplines to study aspects of host-microbial interactions, environmental exposures, health, and the connection to human ecology and sociology. The intersection of these disparate disciplines has necessitated developing a lexicon that pulls from the discipline-specific language used by microbiologists and sociopolitical scientists. Therefore, some authors present their work in a style which is inherent to their primary discipline but novel to microbiome research, which enriches the experience of reading these unique articles. We ask that our readers accept that the language is steeped in scientific acceptance in these fields and read these articles with an open mind.

As with any new form of knowledge production that tries to challenge existing assumptions and introduce new arguments, we understand that some articles might cause discomfort. But if we aspire to allocate microbiology research within a growing field of public health research that is tackling the painful issues of our times, these conversations are necessary and will push our field to a more equitable place.

